# Multiple-trait QTL mapping and genomic prediction for wool traits in sheep

**DOI:** 10.1186/s12711-017-0337-y

**Published:** 2017-08-15

**Authors:** Sunduimijid Bolormaa, Andrew A. Swan, Daniel J. Brown, Sue Hatcher, Nasir Moghaddar, Julius H. van der Werf, Michael E. Goddard, Hans D. Daetwyler

**Affiliations:** 1 Agriculture Victoria Research, AgriBio Centre, Bundoora, VIC 3083 Australia; 2 Animal Genetics and Breeding Unit, University of New England, Armidale, NSW 2351 Australia; 3 NSW Department of Primary Industries, Orange Agricultural Institute, Orange, NSW 2800 Australia; 4 School of Environmental and Rural Science, University of New England, Armidale, NSW 2351 Australia; 5 School of Land and Environment, University of Melbourne, Parkville, VIC 3010 Australia; 6 School of Applied Systems Biology, La Trobe University, Bundoora, VIC 3086 Australia; 7 Cooperative Research Centre for Sheep Industry Innovation, Armidale, NSW 2351 Australia

## Abstract

**Background:**

The application of genomic selection to sheep breeding could lead to substantial increases in profitability of wool production due to the availability of accurate breeding values from single nucleotide polymorphism (SNP) data. Several key traits determine the value of wool and influence a sheep’s susceptibility to fleece rot and fly strike. Our aim was to predict genomic estimated breeding values (GEBV) and to compare three methods of combining information across traits to map polymorphisms that affect these traits.

**Methods:**

GEBV for 5726 Merino and Merino crossbred sheep were calculated using BayesR and genomic best linear unbiased prediction (GBLUP) with real and imputed 510,174 SNPs for 22 traits (at yearling and adult ages) including wool production and quality, and breech conformation traits that are associated with susceptibility to fly strike. Accuracies of these GEBV were assessed using fivefold cross-validation. We also devised and compared three approximate multi-trait analyses to map pleiotropic quantitative trait loci (QTL): a multi-trait genome-wide association study and two multi-trait methods that use the output from BayesR analyses. One BayesR method used local GEBV for each trait, while the other used the posterior probabilities that a SNP had an effect on each trait.

**Results:**

BayesR and GBLUP resulted in similar average GEBV accuracies across traits (~0.22). BayesR accuracies were highest for wool yield and fibre diameter (>0.40) and lowest for skin quality and dag score (<0.10). Generally, accuracy was higher for traits with larger reference populations and higher heritability. In total, the three multi-trait analyses identified 206 putative QTL, of which 20 were common to the three analyses. The two BayesR multi-trait approaches mapped QTL in a more defined manner than the multi-trait GWAS. We identified genes with known effects on hair growth (i.e. *FGF5*, *STAT3*, *KRT86*, and *ALX4*) near SNPs with pleiotropic effects on wool traits.

**Conclusions:**

The mean accuracy of genomic prediction across wool traits was around 0.22. The three multi-trait analyses identified 206 putative QTL across the ovine genome. Detailed phenotypic information helped to identify likely candidate genes.

**Electronic supplementary material:**

The online version of this article (doi:10.1186/s12711-017-0337-y) contains supplementary material, which is available to authorized users.

## Background

Merino sheep are traditionally bred for wool. The value of a sheep’s fleece depends on many characteristics including fleece weight, fibre diameter, staple strength and length, crimp (or curvature), wool color, and dust penetration [1]. Flystrike, particularly around the breech region, is an important disease of Australian sheep, which costs the industry $280 million annually [2]. There are attempts to select for resistance to breech flystrike but direct selection for this trait is not easy to implement in ram breeding flocks because breeding animals are valuable and managed to reduce incidence of flystrike. Several easily assessed or measured indicator traits associated with breech flystrike are available, namely breech wool cover, breech skin wrinkle, dags, wool colour and fleece rot [3, 4].

Genetic variation for these traits is well documented. Estimated heritabilities and correlations for wool traits in Merino sheep are reported in the literature [5, 6]. Genetic correlations between many wool traits including greasy fleece weight and staple length are positive and moderate to high [7] but there are significant antagonisms between some traits. For instance, unfavourable correlations exist between fleece weight and fibre diameter, fibre diameter and staple strength, and wrinkle score and fleece weight [8]. Thus, individual causal polymorphisms are likely to have pleiotropic effects on multiple traits, possibly because they operate through physiological mechanisms that affect wool growth generally. Genetic correlations between measurements at yearling and adult ages for the same trait are moderate to high and range from 0.6 to 0.9 for fleece weights and are higher for fibre diameter [9].

Genomic prediction is an attractive approach for sheep breeders because estimated breeding values (EBV) can be calculated from DNA marker data (genomic selection [10]) when animals are too young to be measured for some phenotypes. In several cases, non-linear Bayesian methods, such as BayesR, result in more accurate EBV than genomic best linear unbiased prediction (GBLUP) because they give more weight to markers that are close to the causal polymorphisms [11].

Genome-wide association studies (GWAS), which use similar data to genomic selection, have been widely used to map causal variants in livestock and humans [12, 13]. Both genomic selection and GWAS are usually performed one trait at a time, which limits their power to detect single nucleotide polymorphisms (SNPs) that are associated with multiple traits and hence to study patterns of pleoitropy. Bolormaa et al. [14] showed that a novel multi-trait analysis, which combines the results from a single-trait GWAS for 56 individual body composition traits in sheep, increased the power to detect pleiotropic quantitative trait loci (QTL). However, precise mapping of QTL may be difficult in such GWAS studies because SNPs that are at a long distance from the QTL can be identified as associated with the QTL due to long-range linkage disequilibrium (LD) between SNPs in livestock. Genomic selection models, especially non-linear models, which fit all SNPs simultaneously, tend to be less affected by this problem, and map causal variants or QTL more precisely than GWAS [11]. Ideally, we would like to find a common set of SNPs with a maximum power to map QTL, to show their pleiotropic effects and to estimate breeding values.

Although multi-trait BLUP genomic selection methods are available, they become cumbersome when the number of traits is very large. Here, we present three approximate multi-trait analyses that use the results from single-trait BayesR and GWAS analyses as data to identify the SNPs that are closest to pleiotropic QTL. We applied these methods for 22 traits (each measured at two ages) that describe wool production and quality (i.e. measured and visually assessed wool traits), and indicator traits associated with susceptibility to breech flystrike on 5726 sheep with genotypes for 510,174 SNPs.

## Methods

### Phenotype data and traits

The Merino and Merino crossbred animals used in this study were sourced from the Information Nucleus (IN) flock of Cooperative Research Centre for Sheep Industry Innovation (Sheep CRC) [15, 16]. In total, 7191 animals were available with phenotype records on 22 traits each measured at two ages (“yearling”; 150 < days < 550 days, and “adult”; ≥550 days), including wool production and quality traits and breech flystrike indicator traits. Trait definitions, numbers of records for each trait, raw means and standard deviations based on the phenotyped animals and number of genotyped animals are in Table 1. A complete description of the design, methods and analyses of wool production and quality assessments is in Hatcher et al. [17].

**Table 1:** Number of records, their mean, standard deviation (SD), estimated heritabilities (h^2^), and variance explained by sire-by-flock interaction for each trait at yearling (Y) and adult (A) ages based on the animals with phenotypic measurements

Trait	Phenotyped animals					Genotyped animals	Trait	Phenotyped animals					Genotyped animals
	Nb	Mean	SD	h^2^	s.f.			Nb	Mean	SD	h^2^	s.f.	
YGFW	5840	3.6	1.06	0.41	0.11	5365	AGFW	4446	5.4	1.77	0.54	0.06	4428
YYLD	5807	71.2	6.46	0.46	0.06	5334	AYLD	4460	74.0	6.02	0.44	0.06	4442
YSL	3859	85.3	15.7	0.62	0.03	3403	ASL	3405	98.9	16.8	0.66	0.04	3399
YSS	3862	30.9	11.6	0.38	0.02	3398	ASS	3397	34.8	10.9	0.38	0.04	3391
YFD	4375	17.3	1.92	0.84	0.04	3915	AFD	3389	18.8	2.70	0.98	0.02	3383
YFDCV	4460	19.3	3.07	0.60	0.02	3999	AFDCV	3425	18.0	2.83	0.55	0.01	3419
YCURV	5353	72.0	12.0	0.63	0.03	5335	ACURV	4437	72.4	12.7	0.72	0.00	4419
YBRWR	5127	2.2	0.95	0.46	0.03	4981	ABRWR	3899	2.2	0.90	0.35	0.06	3884
YBCOV	3826	3.5	0.90	0.22	0.04	3826	ABCOV	2389	3.2	0.98	0.04	0.12	2381
YCCOV	3726	3.5	0.85	0.26	0.05	3724	ACCOV	2418	3.3	0.87	0.37	0.07	2407
YDAG	3956	1.8	1.01	0.09	0.09	3955	ADAG	2762	1.7	0.92	0.04	0.06	2748
YSSTRC	5138	2.7	0.86	0.17	0.14	5127	ASSTRC	3174	2.7	0.94	0.34	0.11	3161
YWEATH	5137	3.1	1.04	0.02	0.16	5126	AWEATH	3174	3.0	1.12	0.13	0.07	3161
YCHAR	5138	2.7	0.86	0.26	0.07	5127	ACHAR	3174	2.7	0.91	0.28	0.05	3161
YFLROT	5027	1.8	1.26	0.20	0.06	5016	AFLROT	3396	1.9	1.43	0.16	0.06	3381
YDUST	5137	3.1	0.98	0.18	0.07	5126	ADUST	3174	2.9	1.15	0.04	0.13	3161
YGCOL	5138	2.5	0.79	0.29	0.08	5127	AGCOL	3174	2.6	0.86	0.20	0.05	3161
YCOLZ	2740	65.6	2.54	0.32	0.00	2738	ACOLZ	2700	65.2	2.42	0.26	0.06	2695
YCOLYZ	2728	8.1	0.78	0.50	0.04	2726	ACOLYZ	2697	8.4	0.77	0.40	0.07	2692
YCOLY	2740	73.8	2.43	0.21	0.00	2738	ACOLY	2708	73.5	2.17	0.19	0.05	2703
YCOLX	2739	69.6	2.24	0.22	0.00	2737	ACOLX	2708	69.4	1.99	0.20	0.04	2703
YSKINQ	1972	2.9	0.72	0.25	0.07	1972	ASKINQ	1798	2.6	0.76	0.07	0.07	1785

GFW = greasy fleece weight; YLD = wool yield; SL = staple length; SS = staple strength; FD = mean fibre diameter; FDCV = fibre diameter coefficient of variation; mean fibre curvature (CURV); BRWR = breech wrinkle; BCOV = breech cover; CCOV = crutch cover; DAG = dag; SSTRC = staple structure, WEATH = staple weathering; CHAR = wool character; FLROT = fleece rot; DUST = dust penetration; GCOL = greasy colour; COL(Z, YZ,Y, and X) = wool clean colour: Z = reflected blue light; YZ = yellowness; Y = brightness; X = reflected red light; SKINQ = skin quality; s.f. = proportion of phenotypic variance explained by sire-by-flock interaction

Prior to shearing at each IN site, the sheep were assessed for a series of visual wool scores, including staple structure (SSTRC), staple weathering (WEATH), wool character (CHAR), fleece rot (FLROT), dust penetration (DUST), and greasy colour (GCOL), and visual breech traits including breech cover (BCOV), crutch cover (CCOV), and dag (DAG) [18]. Each assessment was based on a five-point system in which low scores represent desirable attributes and high scores represent undesirable attributes. A mid-side wool sample (75 to 85 g) was taken from the right side of each animal using an electric handpiece. The samples were measured in a commercial laboratory (AWTA Limited, Melbourne, Vic., Australia) for a range of wool traits. Ten staples from each mid-side sample were randomly sub-sampled to measure staple length (SL) and staple strength (SS). The remainder of each mid-side sample was weighed, washed in hot water with detergent, rinsed in cold water twice, spun and oven-dried at 105 °C. The oven-dried weight was recorded and the 16% regain used to calculate the washing yield (YLD). A Shirley Analyser (AWTA Limited, Melbourne, Vic., Australia) was used to card the dried scoured sample before conditioning at 20 °C and 65% relative humidity for 24 h, after which 2-mm snippets were sampled via mini-coring. The snippets were measured for mean fibre diameter (FD), FD coefficient of variation (FDCV) and mean fibre curvature (CURV) by Sirolan™ Laserscan (AWTA Limited). The washed carded sample was further subsampled and measured for various tristimulus values (T units) that are routinely used to describe aspects of clean colour (X, Y, Z and Y–Z), where X refers to reflected red light, Y to reflected green light and Z to reflected blue light. With wool, the Y value indicates brightness, with increasing values indicating increasing brightness, and the difference between the Y and Z values (Y–Z) indicating wool yellowness [19]. An additional visual breech score, i.e. breech wrinkle (BRWR), was scored post shearing [18]. Not all sheep were measured for all traits.

### Genotype data

This study used the Ovine Infinium^®^ HD SNP BeadChip that was developed under the auspices of the International Sheep Genomics Consortium (http://www.farmiq.co.nz/) and includes 606,006 high-density (HD) SNPs, and the Illumina 50 k Ovine SNP chip (Illumina Inc., San Diego, CA, USA) that includes 54,241 (50 k) SNPs. All SNPs were mapped to the OAR 3.1 build of the ovine genome sequence using SNPchiMp v.3 [20]. Sporadic missing genotypes for the HD SNP chip were filled using FImpute [21]. Quality control of genotypes, imputation of sporadic missing genotypes within each SNP chip, and imputation of the 50 k SNP genotypes to HD SNPs are described in [14, 22]. The details of the quality control are summarised below.

Stringent quality control procedures were applied to the SNP data, i.e. SNPs were excluded if the call rate per SNP (which is the proportion of SNP genotypes that have a GC (Illumina GenCall) score above 0.6) was less than 95%, the minor allele frequency was lower than 0.01 or if departure from Hardy–Weinberg equilibrium (*P* < 10^−5^) was extreme. These criteria were applied on each batch of genotypes separately rather than to the whole dataset. Furthermore, if the average call rate per individual was less than 90%, those animals were removed from the SNP data. The final set of genotyped animals used in this study included 5726 animals with phenotypic records for at least one trait: 690 animals were genotyped for 510,174 SNPs, and the remaining 5036 animals were genotyped with the 50 k SNPs, which were imputed to 510,174 SNPs using a multi-breed population of 1735 animals. Cross-validation within these 1735 HD genotypes revealed an average accuracy of imputation (correlation of imputed empirical non-50 k genotypes) of 0.9871. Most sires of phenotyped animals were genotyped with the HD SNP chip.

### Single-trait genome-wide association studies

Mixed models that fit fixed and random effects simultaneously were used to estimate heritabilities and the effects of SNPs associated with each of the traits were studied. Pedigree heritabilities were estimated based on all animals for which genotype and phenotype data were available. The pedigree file included 10,360 animals (including 785 sires and 3891 dams). The analysis was performed using the ASReml software [23]. The same mixed model was used for the GWAS, except that each SNP (SNP*i*, *i* = 1, 2, 3, …, 510,174) was added to the model as a fixed effect, one at a time, and tested for association with the trait:

1 $${\mathbf{y}} = {\mathbf{1}}_{\text{n}}\upmu + {\mathbf{Xb}} + {\mathbf{s}}_{i}\upalpha_{i} + {\mathbf{Z}}_{{\mathbf{1}}} {\mathbf{a}} + {\mathbf{Z}}_{{\mathbf{1}}} {\mathbf{Qq}} + {\mathbf{Z}}_{{\mathbf{2}}} {\mathbf{s}}.{\mathbf{f}} + {\text{e,}}$$

where **y** is the vector of observed phenotypic values of the animals, **1**_n_ is an n × 1 vector of 1s (n = number of animals with phenotypes), μ is the overall mean, **X**, **Z**_1_, and **Z**_2_ are design matrices relating observations to the corresponding fixed and random effects, **b** is a vector of fixed effects (described below), **a** is a vector of polygenic additive genetic effects sampled from the distribution $N(0,{\mathbf{A}}\upsigma_{\text{a}}^{2} )$, where $\upsigma_{a}^{2}$ is additive genetic variance and **A** is the additive relationship matrix constructed from the pedigree of the animals and their ancestors, **q**, ${\mathbf{s}}.{\mathbf{f}}$, and **e** are the vectors of random effects of breed (including Merino strains), sire-by-flock interaction, and residual error, respectively. **Q** is a matrix with breed and strain proportions calculated from pedigree (*q* ~ $N(0,{\mathbf{I}}\upsigma_{\text{q}}^{2} )$) [24]; **s**_*i*_ is a vector of genotypes for each animal at the *i*th SNP and α_i_ is the corresponding SNP fixed effect. The sire-by-flock interaction effect being significant (*P* < 0.05), it was retained in the model. All models included contemporary group, flock, drop year, sex, birth type, and rearing type as fixed effects and age of measurement, age of dam and its squared value as covariates. Birth type (BT: single = 1, twin = 2, triplet = 3, and quadruplet = 4) and rearing type (RT: single = 1, twin = 2, and triplet = 3) were grouped together (BTRT). The significant interactions between fixed effects (including flock by BTRT, sex by BTRT, and BTRT by age of dam) were fitted in the model. SNPs were tested for significant association with particular traits at several significance thresholds, and the false discovery rate (FDR) [25] was calculated to account for the thousands of significance tests performed. Based on FDR, we chose stringent significance thresholds (*P* < 10^−5^ and *P* < 5 × 10^−7^) to minimise false discoveries (Table 2).

**Table 2:** Number of significant SNPs (*P* < 10^−5^ and *P* < 5 × 10^−7^) and their false discovery rates (FDR, %) for each trait from the single-trait GWAS

Trait^a^	*P* < 1 × 10^−5^		*P* < 5 × 10^−7^		Trait^a^	*P* < 1 × 10^−5^		*P* < 5 × 10^−7^	
	Nb SNPs	FDR	Nb SNPs	FDR^b^		Nb SNPs	FDR	Nb SNPs	FDR^b^
YGFW	69	7.4	7	3.6	AGFW	304	1.7	157	0.2
YYLD	122	4.2	75	0.3	AYLD	113	4.5	59	0.4
YSL	68	7.5	26	1.0	ASL	39	13.1	3	8.5
YSS	56	9.1	31	0.8	ASS	39	13.1	15	1.7
YFD	202	2.5	36	0.7	AFD	295	1.7	69	0.4
YFDCV	97	5.3	31	0.8	AFDCV	109	4.7	47	0.5
YCURV	105	4.9	22	1.2	ACURV	103	5.0	8	3.2
YBRWR	50	10.2	23	1.1	ABRWR	15	34.0	2	12.8
YBCOV	3		2	12.8	ABCOV	6	85.0	1	25.5
YCCOV	19	26.9	1	25.5	ACCOV	45	11.3	19	1.3
YDAG	11	46.4	0		ADAG	5		1	25.5
YSSTRC	41	12.4	2	12.8	ASSTRC	19	26.9	1	25.5
YWEATH	5		0		AWEATH	8	63.8	2	12.8
YCHAR	46	11.1	15	1.7	ACHAR	28	18.2	5	5.1
YFLROT	22	23.2	1	25.5	AFLROT	75	6.8	12	2.1
YDUST	15	34.0	0		ADUST	10	51.0	1	25.5
YGCOL	25	20.4	2	12.8	AGCOL	21	24.3	0	
YCOLZ	28	18.2	5	5.1	ACOLZ	25	20.4	0	
YCOLYZ	7	72.9	1	25.5	ACOLYZ	28	18.2	0	
YCOLY	22	23.2	3	8.5	ACOLY	20	25.5	0	
YCOLX	24	21.3	4	6.4	ACOLX	23	22.2	0	
YSKINQ	9	56.7	0		ASKINQ	7	72.9	0	

^a^Trait names see Table 1

^b^For empty cells, FDR are not available or are higher than 100%

### Genomic prediction

Genomic prediction analyses were performed using two methods: GBLUP [26, 27], with the genomic relationship matrix (GRM) constructed as described by [28] and BayesR [29]. Genomic EBV (GEBV) were estimated in GBLUP directly and calculated from SNP effects in BayesR.

#### Validation populations used for BayesR and GBLUP

The same validation populations were used for both methods. All 5726 genotyped and phenotyped animals were assigned to two groups (straightbred MER and crossbred MER) according to their breed using the breed proportions (**Q**) of animals, which were derived from pedigree [24]. Of the 5726 individuals, 3883 were straightbred MER (Q_MER_ > 0.90) and 1843 were crossbred MER (0.25 < Q_MER_ ≤ 0.90). All crossbred MER animals and the straightbred animals that had sires in common with crossbred MER were in all training sets but not in the validation set. The remaining straightbred MER individuals were split into five sets by allocating all offspring of randomly selected sires to one of the five datasets (fivefold cross-validation approach). In this way, no animal used for validation had paternal half-sibs in the training population. Thus, the analysis was performed five times using each data fold in turn as a validation group and the remaining fourfolds as the training population (i.e. fourfolds plus crossbred MER from above).

#### GBLUP

GEBV were predicted using Model (1), but no single SNP effect (**s**_*i*_) was fitted and **a** was replaced by **g**, where **g** is a vector of GEBV ~$N(0,{\mathbf{G}}\upsigma_{\text{g}}^{2} )$, where $\upsigma_{\text{g}}^{2}$ is the genetic variance and **G** is the genomic relationship matrix (GRM). For a SNP to be included in the GRM, its minor allele frequency had to be higher than 0.005, once genotypes (real and imputed) were combined in the whole dataset. Validation animals were included in the GRM but had unknown phenotypes in the calculation of GEBV.

#### BayesR

The BayesR method [29] assumes that SNP effects are from a mixture of four normal distributions with the variance of each distribution equal to 0, 0.01, 0.1 or 1% of the genetic variance. Gibbs sampling was used to sample from the posterior distributions of the parameters, running 40,000 iterations with 20,000 iterations of burn-in, which were averaged across five parallel chains. Since the BayesR software used in this study does not allow fitting a full-model, residuals were calculated by adjusting the phenotypes for fixed effects, breed effects, and the sire-by-flock interaction effect using ASReml [23]. These residuals were then used as phenotypes in the analysis. The BayesR analysis fits the effects of all SNPs and a residual polygenic effect, the latter with a covariance structure that is proportional to the numerator relationship matrix (**A**). The SNP effects from BayesR were then used to calculate GEBV for animals in the validation sets.

#### Estimation of the accuracy of GEBV

For each validation population, the accuracy of genomic prediction was calculated as the correlation between GEBV and the adjusted phenotype corrected for fixed effects, which was divided by the square root of the heritability of the trait (h^2^) that was estimated by using the 8-generation pedigree of all recorded animals. Thus, we report accuracy as the estimated correlation between GEBV and true breeding values.

### Multi-trait analyses to identify pleiotropic QTL

To identify the pleiotropic genomic regions that control a wide range of wool traits, we used three approximate multi-trait analyses: (1) multi-trait GWAS (multi-GWAS) following the procedure described by Bolormaa et al. [30]; (2) approximate BayesR posterior probability of SNP effects across traits (multi-PP); and (3) the linear combination of local GEBV that were derived from BayesR estimates of SNP effects within 250-kb windows across all traits for a total of 9813 windows (multi-LGEBV).

#### Multi-GWAS

Multi-trait analyses were performed following the procedure in Bolormaa et al. [30] based on SNP effects that were estimated from 44 individual single-trait GWAS. The multi-trait $\chi^{2}$ statistic was calculated as: multi-trait $\chi^{2} = t_{i}^{{\prime }} V^{ - 1} {\mathbf{t}}_{i}$, where **t**_*i*_ is a vector of the signed t-values of the effects of the *i*th SNP for the 44 traits and *V* ^−1^ is the inverse of the 44 × 44 correlation matrix where the correlation is calculated over the 510,174 estimated SNP effects (signed t-values) between each pair of traits. The power of QTL detection was investigated by comparing the FDR [25] from the multi-trait test with the FDR from the single-trait GWAS. To avoid testing a large number of closely-linked SNPs, only the SNPs with the most stringent *P* values (*P* < 5 × 10^−7^) within each 1-Mb window were selected from the multi-trait analysis. These SNPs (*P* < 5 × 10^−7^) were assumed to be near QTL that affect wool traits and were also examined as likely candidate gene positions.

#### Multi-PP

The posterior probability that a SNP had no effect on any trait was calculated as a product of posterior probabilities that a SNP had no effect on any individual trait. We use 1 minus this probability to approximate the probability that a SNP has an effect on at least one trait i.e.: ${\text{pp}}_{{{\text{effect}} \ne 0}} = 1 -\Pi ({\text{pp}}_{{{\text{effect}} = 0}} )$. We retained SNPs with a ${\text{pp}}_{{{\text{effect}} \ne 0}}$ higher than 0.3.

#### Multi-LGEBV

GEBV in the 9813 non-overlapping 250-kb windows (local GEBV) for each animal were calculated based on the BayesR effects of all SNPs in the window. A high variance of local GEBV in a window, means that the window includes a QTL for that trait [11]. For each of the 250-kb windows (segments), the covariance of local GEBV between each pair of the 44 traits was standardized for the variability of each trait as follows:

$$t_{{\left( {y,x} \right)}} = \frac{{\text{cov}_{{LGEBV_{{\left( {y,x} \right)}} }} }}{{\sigma_{y} \sigma_{x} }},$$

where $\text{cov}_{{LGEBV_{{\left( {y,x} \right)}} }}$ is the covariance of local GEBV between trait *y* and trait *x* and $\upsigma_{y}$ and *σ*_*x*_ are the phenotypic standard deviations of trait *y* and *x*. If a window contains a single QTL, we expect this covariance matrix to be dominated by one linear combination of traits representing the QTL effect. Therefore, we carried out a principal component (PC) analysis of the 44 × 44 covariance matrix and examined the highest eigenvalue. This eigenvalue is the variance, in phenotypic standard deviation units, of the linear combination of traits with the largest variance. Across all 9813 windows, the 120 windows with the highest eigenvalues of the first PC (PC1) were arbitrary selected as containing a QTL.

After selecting the 120 top 250-kb windows, we further investigated which SNP, in each of the selected windows, was most highly associated with the PC1 of the (co)variance matrix (the ‘best’ SNP). To identify the best SNP, a pseudo trait (*S*_*LC*_), which consisted of the linear combination of local standardised GEBV and the PC1 eigenvector across the 44 traits, was calculated for each animal at each of the selected 250-kb windows using the following formula: $S_{LC} = y^{{\prime }} x$, where $y^{{\prime }}$ is the transpose of a vector of the local GEBV that are standardised (divided) by the phenotypic standard deviation of each trait (1 × 44) in the corresponding 250-kb window, and *x* is the eigenvector of the PC1 (44 × 1), which was calculated based on the covariance matrix of the standardised local GEBV among 44 traits. Since not all animals were measured for all traits, the missing local GEBV were replaced by the mean of local GEBV across all animals, for which measurements for that particular trait were available in order to calculate a linear combination of the 44 traits. This resulted in a *S*_*LC*_ for each animal in each of the 120 chosen windows, which were now used as a pseudo-trait in GWAS within each window to identify the “best” SNP that tagged variation within the segment. The model used in this GWAS was as follows: $S_{LC} \sim{\text{mean}} + {\text{SNP}}_{i} + {\text{animal}} + {\text{error}}$, where animal and error were fitted as random effects and SNP_*i*_ was fitted as a fixed effect, one at a time (the phenotypes used to calculate *S*_*LC*_ had already been corrected for other fixed effects, breed effect, and sire-by-flock interaction effect prior to BayesR). After performing this new GWAS, the SNP with the highest F value within each of the corresponding 250-kb windows was chosen as the best SNP to tag the QTL.

### Validation of SNP effects

#### Predicting missing phenotypes

The multi-trait validation of SNP effects (i.e. using the linear index approach [30]) requires complete data for all traits at the individual level. For animals without records for a particular trait, missing phenotypes were predicted using a multiple regression approach. This multiple regression used phenotypes that were already corrected for fixed effects, breed effect, and sire-by-flock interaction effect. The multiple regression procedure uses the phenotypic (co)variance matrix between the 44 traits based on all animals (training and validation population), which was estimated using the available phenotypic values. Next, the phenotypic (co)variance matrix was inverted. Then, separately for each animal, traits with phenotypes were ordered before traits with missing phenotypes. Again, for each animal, the missing phenotypes (*y*_*n*_) were then predicted using the following formula:

$$\hat{y}_{n} = - \left( {{\mathbf{U}}^{nn} } \right)^{ - 1} {\mathbf{U}}^{nm} {\mathbf{y}}_{m} ,$$

where **y**_*m*_ is a vector of the traits measured on a particular animal, **U** ^*nn*^ is the inverse of phenotypic covariance matrix between 44 traits with a missing record, and **U** ^*nn*^ is the inverse of phenotypic covariance matrix of the traits with and without a missing record.

#### Validation populations for single-trait and multi-trait analyses

To enable validation of SNP effects in independent animals, the 5726 animals with full phenotypic data (including the predicted phenotypic values) were divided into training and validation populations. The same cross-validation approach was used as described in the genomic prediction section above, except that crossbred animals were not excluded from validation sets. Then, one of the five divisions was randomly used as a validation population and the other four divisions as the training population. Only one 4:1 division (i.e. 4649 training animals: 1077 validation animals, out of 5726 genotyped animals) was tested for each of the traits studied.

#### Validation of SNP effects from the single-trait analysis

The GWAS for all traits were performed separately in the training and validation populations. SNPs with a significant effect in the training population were validated in the validation population for the five traits that displayed the largest number of significant associations. We counted the number of SNPs for which the effect was in the same direction in both the validation and training populations.

#### Selection of top SNPs from each of the three multi-trait methods in the training population

The single-trait BayesR analyses for all traits were also performed using only the training population (4649 animals). Then, the three multi-trait analyses described in the previous section (multi-GWAS, multi-PP, and multi-LGEBV) were repeated in the validation set to validate the top SNPs from each multi-trait analysis in the training population.

#### Use of a linear index in multi-trait validation

The top SNPs in each of the three multi-trait analyses (i.e., three different lists of SNPs) in the training population were validated separately in an independent set of validation animals. A linear index (*y*_*I*_) was calculated for each putative QTL (from each of the three methods) and for each animal. It summarised the information across the 44 traits (22 traits at two ages) and was calculated using the following formula [30]: $y_{I} = b^{{\prime }} {\mathbf{C}}^{ - 1} {\mathbf{y}}$, where $b^{\prime }$ is the transpose of a vector of the estimated SNP effects (not *t* values) on the 44 traits (1 × 44), which were estimated from only the training population, **C** ^−1^ is the inverse of the 44 × 44 (co)variance matrix among the 44 traits calculated from the estimated effects of the 510,174 SNPs, only in the training population, and **y** is a 44 × 1 vector of the phenotype values (adjusted for fixed, breed and sire-by-flock effects) for 44 traits for each animal in the validation sample. This resulted in a linear index (*y*_*I*_) for all putative QTL where, for each QTL, each animal had a linear index summary “phenotype”. Then, GWAS, in which each *y*_*I*_ was treated as a new trait, were performed using the following model:

$$y_{I} = {\text{mean}} + {\text{SNP}}_{i} + {\text{animal}} + {\text{error}},$$

where animal and error were fitted as random effects and SNP_*i*_ was fitted as a fixed effect, one at a time, for significant SNPs (*P* < 10^−5^ for each 1 Mbp) discovered in the training population. After performing the GWAS, the significance (*P* < 0.05) and the consistency of the direction of effects (positive or negative) for the selected significant SNPs were compared between the training and validation populations.

### Identification of the most likely candidate genes

The genes that were located 50 kb upstream and downstream of the best SNP were identified using UCSC Genome Bioinformatics (http://genome.ucsc.edu/) and Ensembl (http://www.ensembl.org/biomart/). If there was more than one gene, we retained only the gene that was located nearest to the SNP or the particular gene with a known effect on wool or hair.

## Results

### Single-trait GWAS

The number of significant SNPs for each trait is in Table 2.

### Genomic prediction

**Fig. 1:**
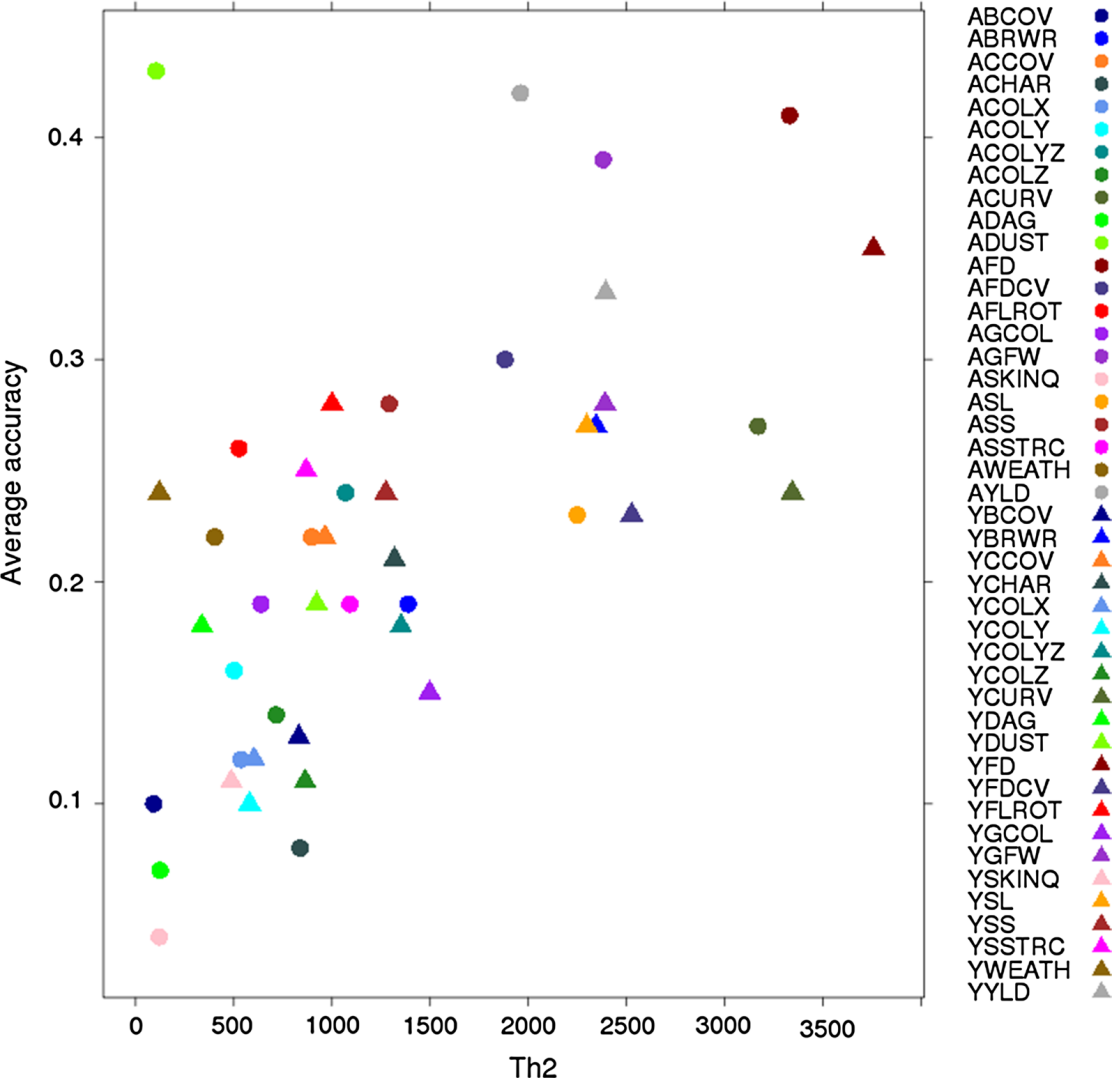
Relationship between BayesR accuracy and number of records (T) multiplied by heritability (h^2^)

**Fig. 2:**
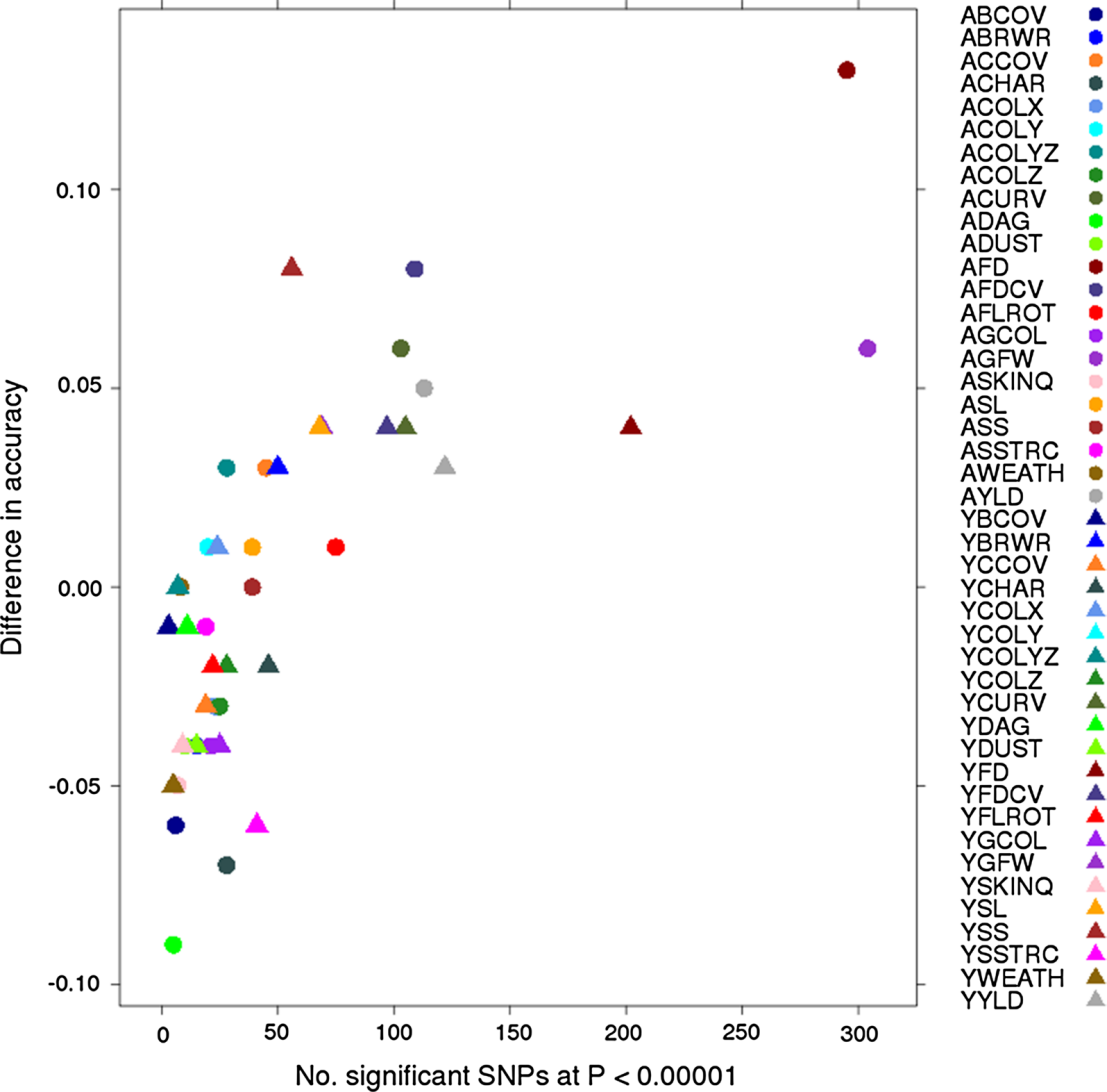
Relationship between number of significant SNPs (*P* < 10^−5^) and difference in accuracy between BayesR and GBLUP

Using BayesR, mean accuracies of genomic prediction of 0.21 and 0.23 were obtained across wool traits at yearling and adult ages, respectively (Table 3). Accuracy tended to increase with Th^2^, with T being the number of phenotypes in the training set (Fig. 1, *R* ^2^ = 0.34). GBLUP provided very similar mean accuracies (0.21 and 0.22 at yearling and adult ages, respectively). However, BayesR tended to result in higher accuracies than GBLUP for traits that had a large number of significant SNPs in the single-trait GWAS (Tables 2, 3; Fig. 2).

**Table 3:** Average accuracies of GEBV of the fivefold cross-validation populations using BayesR and GBLUP methods for each trait at yearling (Y) and adult (A) ages

Trait^a^	Accuracy (SE)		Trait^a^	Accuracy (SE)	
	BayesR	GBLUP		BayesR	GBLUP
YGFW	0.28 (0.011)	0.24 (0.027)	AGFW	0.39 (0.024)	0.33 (0.015)
YYLD	0.33 (0.026)	0.30 (0.021)	AYLD	0.42 (0.042)	0.37 (0.029)
YSL	0.27 (0.024)	0.23 (0.012)	ASL	0.23 (0.033)	0.22 (0.040)
YSS	0.24 (0.026)	0.16 (0.029)	ASS	0.28 (0.028)	0.28 (0.039)
YFD	0.35 (0.015)	0.31 (0.015)	AFD	0.41 (0.033)	0.28 (0.021)
YFDCV	0.23 (0.029)	0.19 (0.021)	AFDCV	0.30 (0.029)	0.22 (0.048)
YCURV	0.24 (0.015)	0.20 (0.009)	ACURV	0.27 (0.018)	0.21 (0.011)
YBRWR	0.27 (0.027)	0.24 (0.018)	ABRWR	0.19 (0.030)	0.23 (0.021)
YBCOV	0.13 (0.035)	0.14 (0.046)	ABCOV	0.10 (0.079)	0.16 (0.054)
YCCOV	0.22 (0.037)	0.25 (0.041)	ACCOV	0.22 (0.042)	0.19 (0.029)
YDAG	0.18 (0.093)	0.19 (0.081)	ADAG	0.07 (0.102)	0.16 (0.090)
YSSTRC	0.25 (0.044)	0.31 (0.045)	ASSTRC	0.19 (0.040)	0.20 (0.032)
YWEATH	0.24 (0.081)	0.29 (0.079)	AWEATH	0.22 (0.098)	0.22 (0.102)
YCHAR	0.21 (0.024)	0.23 (0.019)	ACHAR	0.08 (0.039)	0.15 (0.046)
YFLROT	0.28 (0.055)	0.30 (0.054)	AFLROT	0.26 (0.036)	0.25 (0.041)
YDUST	0.19 (0.051)	0.23 (0.050)	ADUST	0.45 (0.111)	0.49 (0.117)
YGCOL	0.15 (0.011)	0.19 (0.015)	AGCOL	0.19 (0.031)	0.23 (0.040)
YCOLZ	0.11 (0.033)	0.13 (0.018)	ACOLZ	0.14 (0.040)	0.17 (0.037)
YCOLYZ	0.18 (0.030)	0.18 (0.020)	ACOLYZ	0.24 (0.033)	0.21 (0.028)
YCOLY	0.10 (0.038)	0.12 (0.026)	ACOLY	0.16 (0.042)	0.15 (0.050)
YCOLX	0.12 (0.037)	0.11 (0.029)	ACOLX	0.12 (0.069)	0.15 (0.059)
YSKINQ	0.11 (0.032)	0.15 (0.027)	ASKINQ	0.04 (0.091)	0.09 (0.110)
Mean^b^	0.21	0.21	Mean^b^	0.23	0.22

*SE* standard error of average accuracy of GEBV

^a^Trait names see Table 1

^b^Average accuracy across traits

### Multi-trait analyses for the identification of pleiotropic QTL

#### Multi-GWAS

**Fig. 3:**
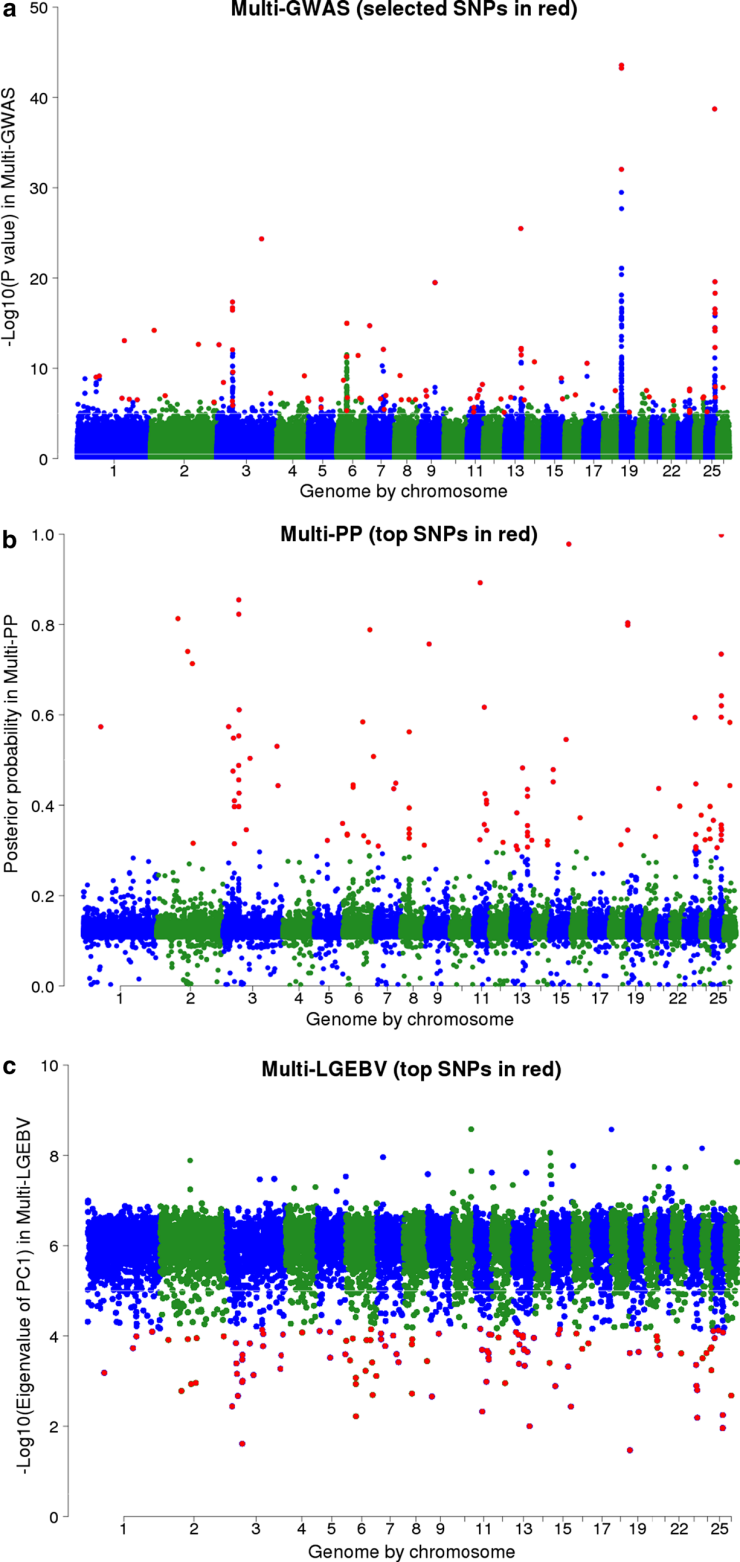
Manhattan plot of multi-GWAS (**a**), multi-PP (**b**), and multi-LGEBV (**c**). *Y* axes are −log_10_ (*P* values) of SNPs for multi-GWAS, multi-trait posterior probabilities for multi-PP, and eigenvalues (×1000) of the first principal component (PC1) of 9813 250 kb-windows for multi-LGEBV. Numbers on the *x* axes represent the number of ovine chromosomes (OAR) excluding OARX. SNPs in *red colour* represent the top selected SNPs from each of the three multi-trait analyses [the highest eigenvalues of 120 windows from multi-LGEBV, multi-trait posterior probabilities of 102 SNPs from multi-PP, and 102 multi-GWAS SNPs including 64 top SNPs (*P* < 5 × 10^−7^) in 1-Mb intervals and 38 SNPs (*P* < 10^−5^)]

**Fig. 4:**
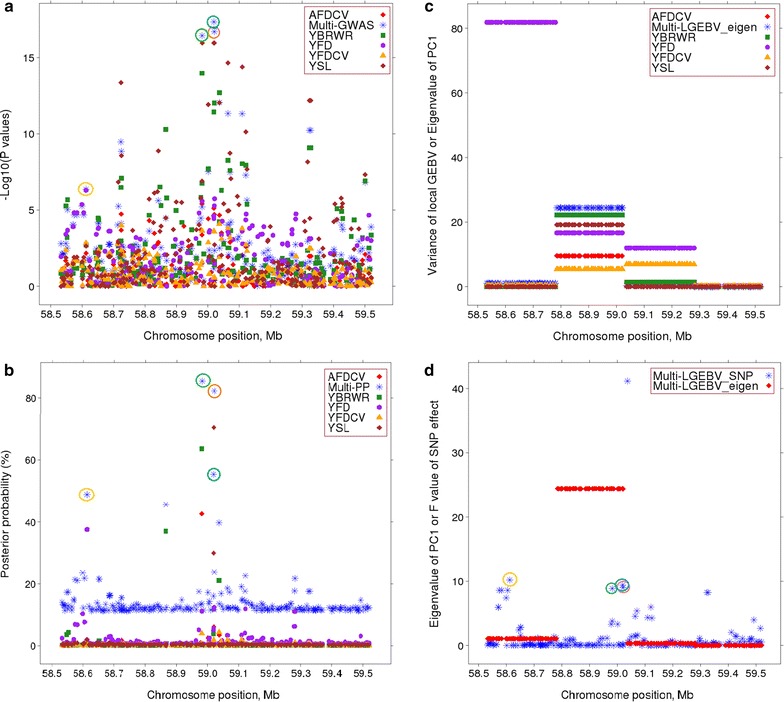
Plots of various mapping approach statistics for the OAR3 region between 58.5 and 59.5 Mb. −log_10_ (*P* values) of SNP effects of five single-trait GWAS and multi-GWAS (**a**), posterior probabilities of SNP for five single-trait BayesR and multi-PP (**b**), variance of local GEBV in 250-kb intervals for five traits (arbitrarily scaled) and eigenvalues of PC1 (×10^3^) of multi-LGEBV (**c**), and eigenvalues of PC1 (×10^3^) from multi-LGEBV and F values of SNP effects (×10^−3^) for PC1 (**d**). SNPs *circled in green* and *orange* are the two top SNPs from each of the three multi-trait methods in that particular window and the top SNPs in the adjacent window are *circled in yellow*

The multi-trait analyses using GWAS identified many narrow regions that contained more than one significant SNP (e.g. on chromosomes OAR3, 6, 7, 13, 19, and 25, OAR for *Ovis aries* chromosome; Fig. 3a). Combining the single-trait GWAS in a multi-trait analysis resulted in 563 and 263 significant SNPs at significance thresholds of *P* < 10^−5^ and *P* < 5 × 10^−7^, respectively. This corresponded to a FDR of 0.9 and 0.1% (respectively), which was lower than for any individual trait tested in the single-trait GWAS (Table 2). In order to avoid testing a large number of closely-linked SNPs, we identified 64 SNPs that were significant at *P* < 5 × 10^−7^ and which were separated from each other by at least 1 Mb. Figure 4a compares the multi-GWAS with five single-trait GWAS for a region around 59.0 Mb on OAR3. These five traits (AFDCV, YBWR, YFD, YFDCV, and YSL) were those for which the number of significant SNPs (*P* < 5 × 10^−7^) was largest in the single-trait GWAS (Table 2).

#### Multi-PP

One hundred and two SNPs had an approximate multi-trait posterior probability (pp) higher than 0.3 (Fig. 3b), among which two SNPs had a ${\text{pp}}_{{{\text{effect}} \ne 0}}$ higher than 0.05 for four traits, 11 for three traits, 34 for two traits and the remaining 55 SNPs for one trait. Thus, although the multi-trait pp was calculated using all traits, the pp for the 102 identified SNPs was mainly influenced by between one and four traits. For example, Fig. 4b compares the multi-pp of SNPs on OAR3 around 59.0 Mb with the pp for the five traits YSL, YFD, YFDCV, AFDCV, and YBRWR. Two SNPs that were 39 kb apart had a high pp i.e. higher than 0.82 (Fig. 4b) and were in high LD (*r* ^2^ = 0.44), which indicates that they may tag the same QTL. There is another SNP with a multi-trait pp ≈ 0.5 that is located 0.5 Mb upstream of these two SNPs.

#### Multi-LGEBV

Local GEBV, using only the SNPs within a 250-kb window, were calculated for each animal using the BayesR estimates of SNP effects. The variance of local GEBV for each window and trait was calculated. A high variance indicates that within the 250-kb window there is a QTL for that trait. The highest percentage of variance of local GEBV was equal to 1.9% of the phenotypic variance for yearling staple length (YSL), which indicates that we did not detect QTL with very large effects for any of the traits. Figure 4c shows the variance for five traits for four windows around OAR3:59 Mb.

**Fig. 5:**
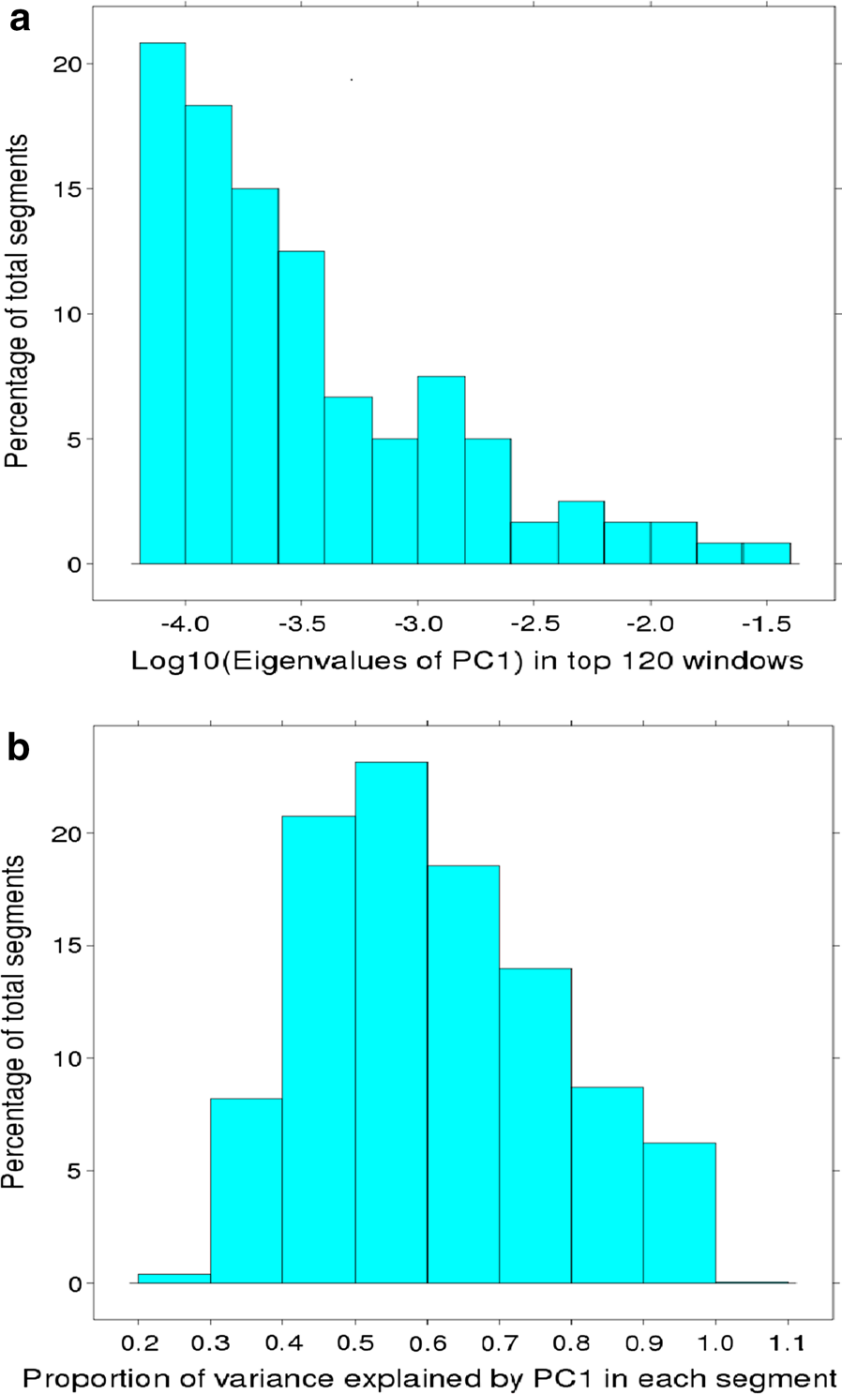
Log_10_ of the highest 120 PC1 eigenvalues (**a**) and proportion of variance explained by PC1 across 9813 windows (**b**)

For each of the 9813 windows, a PC analysis yielded 44 eigenvalues and eigenvectors. A high first eigenvalue indicates a window that contains a QTL. We selected the 120 windows with the highest eigenvalues as windows that potentially contain a QTL (Fig. 3c). The distribution of the log_10_ of the first eigenvalues for 120 segments with the highest first eigenvalue is in Fig. 5a. If within the window, there is a single QTL that has an effect on multiple traits, we expect that the first eigenvector will explain most of the variance and that the other eigenvalues will be low. We selected 120 segments with the highest first eigenvalues across all 9813 segments and in 112 of these 120 selected segments, the PC1 explained more than 90% of the total variance. The distribution of the proportion of variance explained by PC1 eigenvalues across the 9813 windows is in Fig. 5b.

Figure 4c shows the first eigenvalues for four segments on OAR3. The second segment (58.75–59.25 Mb) had the highest eigenvalue, but the segment to its left had a high variance of local GEBV for YFD in spite of a lower first eigenvalue. This suggests that the first segment contains a more pleiotropic QTL while the second segment contains a QTL that affects YFD mainly.

In order to identify the SNP that is closest to the QTL (best SNP), for each of the 120 segments, we performed a new GWAS by using the eigenvector of PC1 as a new trait and fitting each SNP, one at a time, within each segment. Thus, the dependent variable was the linear combination of traits as defined by the first eigenvector. By this process, we selected the top SNPs (with the highest F value) in each of the top 120 segments. For example, Fig. 4d shows the eigenvalues (×10^3^) of the four neighboring windows at around 59.0 Mb on OAR3 and the F values of the SNP effects (×10^−3^) for PC1 in the corresponding windows. The second segment had a high eigenvalue that explains 98% of the total variance and three SNPs (circled in blue, orange and green) were most highly associated with the first eigenvector for this segment. The first segment had a comparably lower first eigenvalue but this eigenvalue explained 96% of the total variance and there is only one SNP (circled in yellow) that was associated with the first eigenvector for that segment. The third segment had an even lower first eigenvalue (explaining 68% of the total segment variance), which probably indicates that there is no QTL in that segment. However, we did observe one SNP that was highly associated with the first eigenvector (Fig. 4d), which illustrates one of the drawbacks of this method. Since local GEBV are calculated from SNP genotypes, there will always be one or more SNPs associated with the first eigenvector but it should not be interpreted as evidence for a QTL unless the first eigenvalue is high.

#### Comparison and combination of results from the three multi-trait analyses

**Fig. 6:**
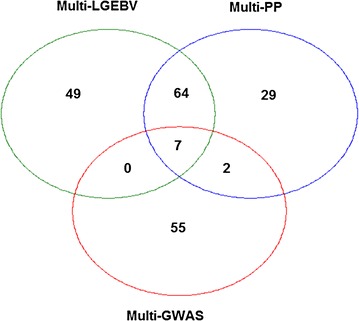
Venn diagram of the overlapping SNPs between the top SNPs selected by the three multi-trait analyses

As described above, we selected 64 SNPs from the multi-GWAS (most significant SNP (*P* < 5 × 10^−7^) in each 1-Mb window), 102 SNPs with the highest multi-PP and 120 from the analysis of local GEBV. Among these, 75 SNPs overlapped across two or three analyses. Therefore, the total number of SNPs identified was 206. Of these 206 SNPs, seven were identified by all three methods. In addition to these seven SNPs, 64 were identified by both local GEBV and multi-PP, and two by multi-PP and multi-GWAS (Fig. 6 and see Additional file 1: Table S1). Of the 64 top multi-GWAS SNPs, 55 were not among the top SNPs selected by the other two multi-trait methods. In fact, 50 of these 55 SNPs were in segments that did not have a high eigenvalue based on local GEBV, and the first eigenvalue explained less than 90% of the total variance. Thus, it is possible that these SNPs are at some distance from the causal variant, which is located in another segment. Conversely, among the SNPs that were identified by the multi-LGEBV and multi-PP analyses, 38 (=9 + 13 + 16) were significant in the multi-GWAS (*P* < 10^−5^) although they were not the most significant SNPs in their 1-Mb window, or failed to reach a *P* < 5 × 10^−7^. By adding these 38 SNPs to the 64 SNPs from the multi-GWAS, 20 (=7 + 13) are common to all three multi-trait approaches, nine in the multi-LGEBV and multi-GWAS and 18 (=2 + 16) in the multi-PP and multi-GWAS (Fig. 6).

**Fig. 7:**
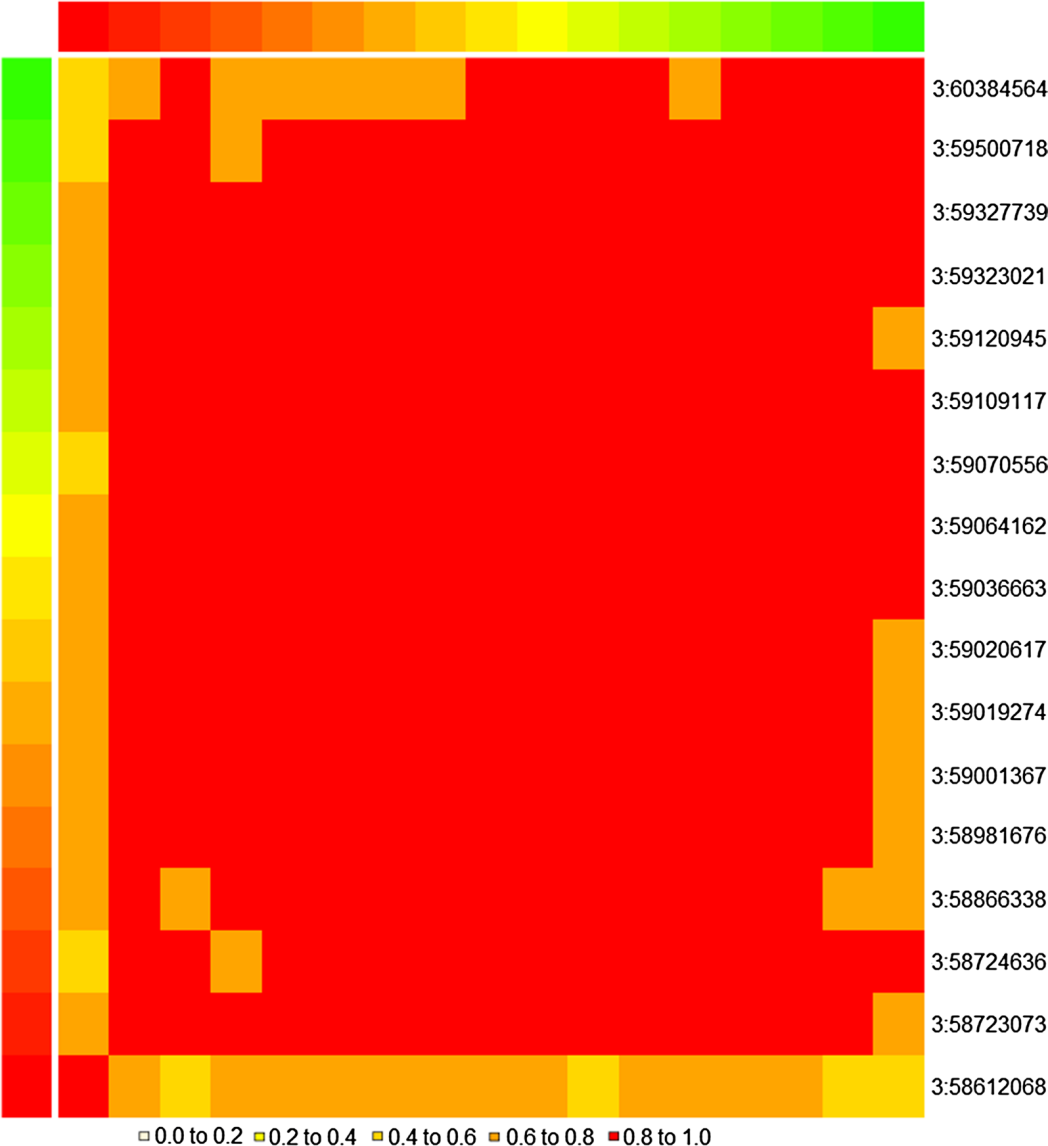
Correlation matrix of the effects on 44 traits between the 16 top SNPs within the 58.5–59.5 Mb region on OAR3. *Numbers on the right* represent chromosome number and position in base pairs of these top SNPs

**Fig. 8:**
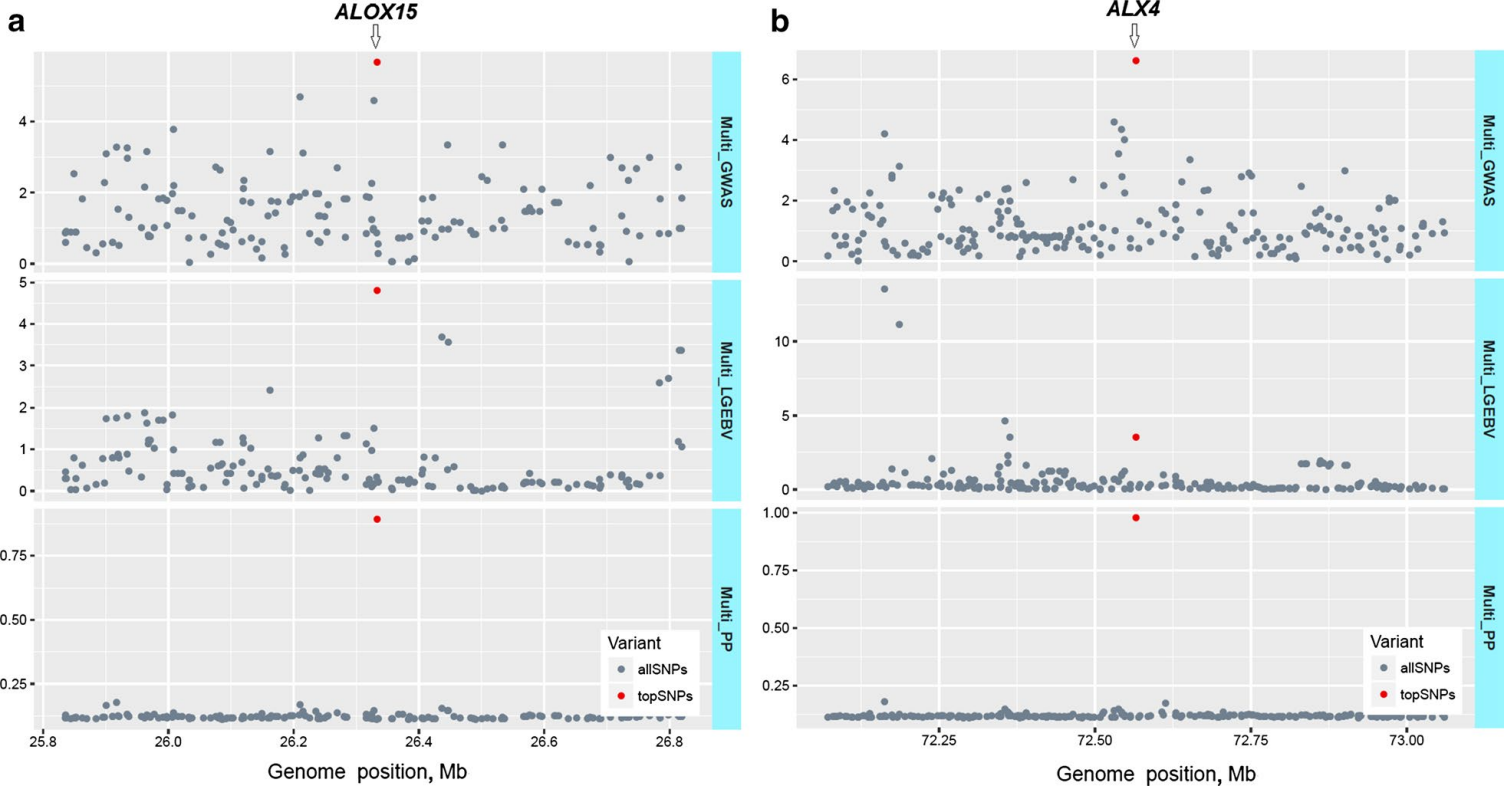
SNP effects estimated by three multi-trait analyses (multi-GWAS, multi-PP, and multi-LGEBV). **a** On OAR11 near the *STAT3* gene. **b** On OAR15 near the *ALX4* gene. The top SNPs identified by these three methods are indicated by a *red circle* on each plot. Note that the scale of the *y* axis is different for each graph. The scale for multi-GWAS is −log_10_ of the *P* values to the corresponding F values of SNP effects, for multi-LGEBV is −log_10_ of P values to the corresponding of F values of SNP effects for PC1, which was divided by 1000, and for multi-PP is multi-trait ${\text{pp}}_{{{\text{effect}} \ne 0}}$

These results are illustrated in Fig. 4. The points that are circled in the same colour in Fig. 4a, b, d represent the same SNP. The second segment had the highest eigenvalue and explained 98% of the total variance of local GEBV in that segment. The SNPs circled in blue, orange, and green are those that were most highly associated with the first eigenvector (Fig. 4d) and had the highest multi-PP (Fig. 4b). These were also highly significant in the multi-GWAS (Fig. 4a). The SNP circled in blue (at 59,019,274 bp on OAR3) was identified by all three multi-trait methods (see Additional file 1: Table S1). In all three methods, the same traits (YSL, YBRWR, YFDCV, AFDCV) contributed to the multi-trait statistics, which supports the conclusion that this is a pleiotropic QTL with effects on at least these four traits. The first segment in Fig. 4 had a high variance of local GEBV for YFD, but when this trait was combined with the other 43 traits, the first eigenvalue was lower than that of the second segment. However, the SNP that is circled in yellow was associated with the first eigenvector, had a high multi-PP and was significant (*P* < 10^−5^) in the multi-GWAS. In all three analyses, this SNP was associated with YFD. In Bayes R, which generates the statistics for multi-PP and local GEBV, all SNPs are fitted simultaneously in the model. Thus, it is possible that the QTL tracked by the yellow circled SNP differed from the QTL tracked by the blue, orange and green circled SNPs. This hypothesis is supported by the traits that are affected by the SNP. Using the effect of each SNP on the 44 traits as estimated by the GWAS, it is possible to calculate the correlation between any pair of SNPs. Figure 7 displays the correlation among SNP effects in this region of OAR3 as a heat map. It shows that all the other SNPs except that circled in yellow have highly correlated effects and presumably track the same QTL. In the multi-GWAS (Fig. 4a), another significant SNP at 58.72 Mb was observed, which was not detected in either of the other analyses presumably because it tracked the same QTL as the SNP at 59.0 Mb circled in blue, orange and green colours. Figure 8 illustrates two more straightforward examples where the same SNP was identified by all three methods.

### Validation of SNP effects

#### Validation of SNPs from single trait GWAS

The most significant SNPs (*P* < 10^−5^) in each 1-Mb window were tested in the validation population. Table 4 shows the results for five traits, which were among those that had the largest number of significant associations (Table 2). The number of SNPs tested ranged from 14 (YYLD) to 101 (YFD). The proportion of these SNPs that were significant at *P* < 0.05 in the validation population varied from 0.07 (=1/14 for YYLD) to 0.30 (=7/23 for AYLD). The percentage of these SNPs that had an effect in the same direction in both the validation and training populations varied from 57 to 86% (Table 4). In addition, when a significance level was also imposed on SNPs discovered in the validation population, the percentage of SNP effects in the same direction across training and validation populations increased from 75 to 100% (small numbers of SNPs were discovered in the relatively small validation population partly due to lack of power).

**Table 4:** Number of significant SNPs (*P* < 10^−5^) in each 1-Mb region in the training population that were also significant in the validation population for the five individual single traits at two yearling (Y) and adult (A) ages

*P* value^a^	Number of SNPs	%-same	*P* value^a^	Number of SNPs	%-same
*YGFW*			*AGFW*		
0.05	2	100	0.05	7	100
All	20	75	All	41	80
*YYLD*			*AYLD*		
0.05	1	100	0.05	7	86
All	14	57	All	23	61
*YSL*			*ASL*		
0.05	3	100	0.05	4	75
All	24	67	All	27	74
*YFD*			*AFD*		
0.05	24	100	0.05	15	93
All	101	86	All	92	78
*YCURV*			*ACURV*		
0.05	6	100	0.05	2	100
All	30	83	All	28	82

%-same = percentage of SNPs, which have an effect in the same direction in both the training and validation populations

^a^ *P* value in the validation population

#### Multi-trait validation using the linear index approach

Association between a SNP and its corresponding linear index was tested in the validation sample. The 105, 77, and 120 top SNPs were selected from the multi-GWAS (*P* < 5 × 10^−7^ in each 1-Mb window), multi-PP, and multi-LGEBV analyses, respectively, in the training population. These SNPs were tested in a GWAS in the validation population (see Table 5). Of the 105 multi-GWAS SNPs that were significant in the training population, 70% had an effect in the same direction in both the training and validation populations and 16 were also significant (*P* < 0.05) in the validation population of which 88% had an effect in the same direction in both the validation and training populations (Table 5). The results were slightly better for the 77 SNPs selected by the multi-PP analysis: 19 out of these 77 SNPs were significant and all had an effect in the same direction in both the validation and training populations. Thus, the number of validated SNPs (with a significant effect in the same direction) was largest with the multi-PP approach (19) followed by multi-LGEBV (15) and multi-GWAS (14 = 16 × 0.88).

**Table 5:** Validation of the top SNP effects from three multi-trait analyses

*P* value^a^	Number of SNPs	FDR %	%-same
*SNPs from multi*-*GWAS*			
0.05	16	29.3	88
All	105		70
*SNPs from multi*-*PP*			
0.05	19	16.1	100
All	77		75
*SNPs from multi*-*LGEBV*			
0.05	15	36.8	100
All	120		74

%-same = percentage of SNPs, which have an effect in the same direction in both the training and validation populations

^a^ *P* value in the validation population

### Examples of QTL with a similar pattern of effects across traits

If two QTL affect the same physiological pathway, one might expect that they have the same pattern of effects. We assessed the similarity of SNP effects by the correlation between pairs of SNPs across the 44 traits. Two SNPs might have a similar pattern because they affect the same pathway or because they tag the same QTL. Among the 206 SNPs, strong correlations (>0.8) were mainly found among the SNPs located on the same chromosome. For instance, six SNPs were on OAR3:59.0 Mb, three on OAR5:48.5 Mb, four on OAR6:37.5 Mb, five on OAR8:31.2 Mb, six on OAR13:62.9 Mb, three on OAR19:0.6 Mb, five on OAR23:44.3 Mb, and 11 on OAR25:35.3 Mb. Some of these were in high LD (e.g. LD estimates (*r* ^2^) between three SNPs on OAR3 and OAR19 ranged from 0.50 to 0.95). It is likely that each of these clusters of highly correlated SNPs tag one major QTL. In a few cases, moderate correlations (0.6 to 0.8) existed between SNPs located on different chromosomes. For instance, the SNP at OAR3:59.0 Mb (near the *FOXI3* gene) has a similar pattern of effects as the SNP at OAR6:37.5 Mb (near *LCORL*) and OAR23:44.2 and OAR25:35.3 (near *MAT1A*). In each case, there is an allele that increases SL, SS and FD, but decreases FDCV. However, the SNPs differ in their effects on other traits, such as GFW, thus it is less likely that they act through the same pathway. Similarly, the SNP at OAR13:62.9 (near *RALY*) and OAR15:72.6 (near *ALX4)* both have an allele that increases FD and FDCV.

### Identification of candidate genes

We searched for genes within 50-kb genomic regions up and downstream from each of the 206 SNPs selected from the three multi-trait analyses (see Additional file 1: Table S1) and identified many genes with known effects on wool and hair growth (Table 6). We also found strong effects for SNPs near the genes *ALOX15*, *ANKS1B*, *ELOVL6*, *FASN*, *NCAPG*, *LCORL*, *FTL*, and *GK5*, which are associated with growth, fatness and body composition in sheep and humans [14, 31–33].

**Table 6:** List of candidate genes with known effects on wool or hair growth

Gene code	Start and stop position of gene (bp)	Gene function	References
*ITGA6*	OAR2:136,225,512–136,275,286	Surface markers for epithelial stem cells within hair follicles	[56, 57]
*ANTXR1*	OAR3:38,977,681–39,235,539	Affects hair follicle growth and cycling; Alopecia	[58, 59]
*FOXI3*	OAR3:58,986,758–58,990,671	Regulates multiple aspects of hair follicle development and homeostasis	[47]
*KRT86*	OAR3:133,936,440–133,944,796	Maintains strength and elasticity of hair	[59, 60]
*WNT1* ^a^	OAR3:137,053,186–137,056,187	Wnt/β-catenin signalling is necessary for hair follicle stem cell proliferation	[61]
*BMPER*	OAR4:62,662,041–62,917,582	BMP signalling controls the hair follicle cycle	[49]
*FRAS1*	OAR6:92,393,951–92,908,337	Basement membrane protein; dermal-epidermal adhesion	[62]
*FGF5*	OAR6:94,584,400–94,605,575	Induces regression of the human hair follicle; regulator of hair growth	[63]
*FGF7*	OAR7:57,779,972-57,841,735	Regulates cell proliferation and cell differentiation and is required for normal regulation of the hair growth cycle	[64]
*STAT3*	OAR11:41,903,051–41,934,839	Keratinocyte stem homeostasis; alters behaviour of hair follicle stem populations	[65]
*OVOL2* ^a^	OAR13:37,399,961–37,424,537	Controls epithelial cell proliferation and differentiation in hair bulb and skin	[66]
*EIF2S2*	OAR13:62,907,171–62,923,869	Inhibition of eIF4E protects against cyclophosphamide-induced alopecia	[67]
*ALX4*	OAR15:72,556,058–72,606,253	Affects hair follicle growth and cycling; total alopecia (hair loss)	[59, 68]
*MAML2*	OAR15:13,755,425–13,774,117	Modifies Notch signalling that controls a cell fate switch in hair follicle stem cells	[69]
*CUX1*	OAR24:34,631,867–34,950,962	Essential for epithelial cell differentiation of the hair follicle in mice	[70]
*ACHE* ^a^	OAR24:35,649,479–35,653,376	M4 muscarinic acetylcholine receptors play a key role in the control of murine hair follicle cycling and pigmentation	[71]

^a^Not the nearest gene to the particular SNP with a significant effect, but is a gene with a known effect on wool or hair

## Discussion

### Genomic prediction

Our results show that the estimated GEBV accuracies are affected by trait, size of the training population, and statistical method used (GBLUP vs. BayesR). As expected from theory [34] in which Th^2^ is a critical parameter, traits with a high heritability and a large training population tended to result in higher accuracies than those with an average heritability across populations (Fig. 1).

On average, BayesR and GBLUP resulted in similar GEBV accuracies but BayesR resulted in higher accuracy for traits (GFW, YLD, SL, SS, FD, FDCV, and CURV) for which there was a large number of significant SNPs in the GWAS. In an earlier study on the genomic prediction of wool and carcass traits using the 50 k SNP chip based on the same population, Daetwyler et al. [22] found no clear differences in accuracy between GBLUP and BayesR. Kemper et al. [11] found that accuracy of across-breed genomic predictions for selection candidates that are less related to the training animals was higher with BayesR than with GBLUP and the use of BayesR mapped QTL more accurately than GBLUP in dairy cattle. Many other studies in cattle and humans have reported that BayesR results in more accurate GEBV than GBLUP, in particular for traits for which mutations of moderate effect are segregating [29, 35–37].

There are few reports on the accuracy of genomic predictions in sheep [38–43]. Pickering et al. [43] reported the accuracy of genomic predictions for health traits including dagginess for several New Zealand breeds (Romney, Coopworth, Perendale, Texel and three breed crosses). For the dagginess traits, they found that accuracies of genomic predictions ranged from 0.11 to 0.56 for those breeds, while in our study an accuracy of 0.19 was estimated for YDAG using GBLUP. Daetwyler et al. [38] (on the same population as used here but with fewer records) reported that accuracies of GEBV with 50 k SNPs ranged from 0.15 to 0.79 for wool traits in Merino sheep and from −0.07 to 0.57 for meat traits in all breeds studied. These accuracies were higher than those that we obtained for wool traits. Several factors may explain the difference between our results and those in these previous studies: (1) only 107 animals were included in their validation set resulting in accuracies with larger standard errors; (2) some animals in their validation set were closely related to animals in the training dataset, whereas we deliberately limited relationships between training and validation animals; and (3) we used within-strain GEBV (i.e., adjusted for genetic differences between Merino strains) whereas they computed GEBV accuracy in the overall population.

Our validation approach, in which the relationship between training and validation populations is minimized, is relevant to the commercial use of genomic selection in which a sheep breeder relies on a training population that is not closely related to his own sheep. In our case, the average of the top 10% genomic relationships that a validation individual has with animals in the training population was equal to 0.02. When validation animals are more related to the training animals, it is likely that the estimated genomic prediction accuracies will be higher.

The level of genomic prediction accuracy may also depend on the accuracy of imputation [44]. However, based on the imputation tests from 50 k to HD SNPs (not reported here), the mean imputation accuracy between imputed and real non-50 k genotypes on our HD data (as the proportion of correctly imputed genotypes for non-HD SNPs) was higher than 0.98, which means that this is not a likely reason for the low prediction accuracies obtained with our data.

Overall, the accuracy of GEBV was low. The similar accuracies obtained with GBLUP and BayesR suggest that there are few QTL of moderate or large effect, which is supported by the single-trait GWAS results for many traits. Therefore, we investigated whether combining information from all traits could help to identify QTL for multiple traits.

#### Multi-trait analyses

It is known that genetic correlations exist between many of the traits studied here and thus there must be QTL with effects on multiple traits. We used three multi-trait analyses to map these QTL. All three methods involve a degree of approximation, thus it is difficult to apply precise significance tests. However, the fact that there is agreement among the results of the three multi-trait and with the single-trait results supports the conclusion that they detect pleiotropic QTL. This is also supported by the rates of validated individual SNPs.

The multi-trait GWAS method used here was previously shown to increase the power of QTL detection [14, 30]. In most 1-Mb intervals that we selected, the pattern of the SNP effects is similar across traits, thus the high correlation among traits, which implies that there is probably only one major QTL within a given interval. The most significant SNP in a region varies from one trait to another due to sampling error even if there is only one QTL. Unless the errors for different traits are highly correlated, the multi-trait analysis reduces the sampling error, which results in a more precise localization of the QTL. However, because long range LD occurs in the ovine genome [45] a SNP that is located at a long distance from any QTL can still have a significant association with the trait. Combining this with the large number of QTL that affect most complex traits, a SNP associated with one QTL may merge with those associated with another nearby QTL, which decreases our ability to map the QTL. To overcome this difficulty, methods that fit all SNPs simultaneously, such as the BayesR method [29] used here, have been advocated for QTL mapping.

In dairy cattle, Kemper et al. [11] showed that BayesR maps QTL more precisely than GWAS. A multi-trait BayesR analysis with 44 traits would impose a very large computational burden, thus we used two approximate methods (multi-PP and multi-LGEBV) to combine the results from 44 single-trait BayesR analyses. In the single-trait BayesR analysis, high variance of local GEBV indicates the presence of a QTL in that 250-kb window. The equivalent multi-trait test is based on the first eigenvalue that is caused by one or more traits having a high variance of local GEBV. If there is more than one trait with a high variance and if the local GEBV are correlated between different traits (as expected if there is only one QTL), the first eigenvalue increases. Windows with more than one QTL can occur but for 112 of the 120 windows with the highest eigenvalues, the first eigenvalue explained more than 90% of the total variance, which indicates that windows with only one QTL predominated in our study. To identify the SNP that was located nearest to this QTL, we carried out a local GWAS using the local GEBV as a new trait.

Since multi-LGEBV and multi-PP both used the output from single-trait BayesR analyses, it is reassuring that they detected many common SNPs. In fact, of the 102 SNPs found by multi-PP, 64 were also detected among the 120 best SNPs from the multi-LGEBV (Fig. 6). However, there was less agreement between multi-GWAS and the BayesR based methods. In particular, when we considered only the top multi-GWAS SNPs (*P* < 5 × 10^−7^) in each 1-Mb window, the multi-GWAS detected 64 SNPs, but only seven were found by the other two methods. Fifty-five of the SNPs detected by the multi-GWAS were not identified by the other two methods but 50 of them are located in the windows, which had a low eigenvalue of PC1 with PC1 explaining less than 90% of the total variance. Decreasing the threshold for the multi-GWAS to *P* < 10^−5^ (Fig. 6) improves the agreement between the multi-GWAS and the other two methods.

### Validation of SNP effects

The proportion of SNPs that were confirmed in the validation set by the multi-trait methods was equal to that obtained for the best single trait (fibre diameter) and higher than that for most single traits. Among the three multi-trait methods, multi-PP had the highest validation rate. This was unexpected since the two other methods used GWAS in both training and validation, whereas multi-PP did not use GWAS methods in the discovery process. However, a multi-PP can miss some QTL since it relies on finding individual SNPs with a high posterior probability. In some cases, the evidence for a QTL is spread across many SNPs each with a low posterior probability, and thus the local GEBV variance is more likely to detect the QTL than the posterior probability.

### Identification of QTL with similar patterns of pleiotropic effects

Stearns et al. [46] pointed out that the relative advantage of multivariate over univariate approaches varied with the level of genetic covariance between traits. In this study, some of the wool traits are genetically highly correlated. Previously, Bolormaa et al. [14] used a correlation matrix of pairs of SNP effects across 56 meat and body composition traits to perform a hierarchical clustering analysis. Using this approach, they identified at least four groups of QTL with similar patterns of pleiotropic effects on body composition (the population of sheep was similar to that used here). In our study on wool traits, the clustering analysis based on 206 SNPs was also done using GWAS SNP effects, but there were no clear-cut clusters of SNPs (results not shown).

### Candidate genes

By exploiting pleiotropic effects for mapping QTL, we identified 206 putative QTL, which were close to 130 genes (within a distance of 50 kb on either side of each SNP). In some cases, the known function of the candidate gene fits the observed phenotype well. Table 6 provides a list of candidate genes with known effects on wool or hair growth. For instance, SNPs that are located around 59.0 Mb on OAR3 and near the *FOXI3* gene, are associated with multiple traits including wool quality and breech conformation traits (FDCV, SL, SS, FD, BRWR, BCOV, CCOV, and FLROT) at both ages. *FOXI3* regulates multiple aspects of hair follicle development and homeostasis and loss of *FOXI3* impedes hair follicle down-growth and progression of the hair cycle [47]. Shirokova et al. [47] showed that *FOXI3* displays a highly dynamic expression pattern during hair morphogenesis and cycling. In mice, absence of *FOXI3* results in a sparse fur phenotype and poor hair regeneration after hair plucking, and these effects are exacerbated with age due to impaired secondary hair germ activation leading to progressive depletion of stem cells. A SNP at 62.6 Mb on OAR4, which is located near (9.8 kb) the *BMPER* gene, was detected through multi-LGEBV and multi-GWAS (*P* = 2 × 10^−4^). Bone morphogenetic protein (BMP) signalling regulates hair follicle cycle and development [48–51]. BMP signalling is a critical feature of the complex epithelial–mesenchymal cross-talk necessary to produce hair [50].

We also found significant SNPs (Table 7) close to genes (*ALOX15*, *ELOVL6*, *FASN*, *NCAPG*, and *GK5*) that are associated with variation in size, fatness and body composition in sheep, cattle and humans [7, 14, 32, 33]. This is not surprising since greasy fleece weight and fibre diameter have positive genetic correlations with yearling weight (0.23 and 0.17, respectively; [7]). The SNPs that are located within or near (<5 kb) *ALOX15*, *FASN*, *FTL*, and *NCAPG* were associated with GFW (up to |t| = 8.9), while the SNPs in *ELOVL6* and *GK5* had significant associations with FD (up to |t| = 4.7). Another SNP with an effect of |t| = 4.3 for GFW is located at 37,559,817 bp on OAR6 with the nearest gene being *LCORL* (at 107 kb). Not surprisingly, the effects of SNPs in *NCAPG* and near *LCORL* were highly correlated (r > 0.8) in our data, which indicates that there may be only one QTL in this region [52–54]. Furthermore, we found that the *LCORL* SNP identified in our study (at OAR6:37,559,817 bp) is located 29 kb from the *LCORL* SNP that was detected in a multi-trait GWAS across carcass and growth traits (at OAR6:37,530,647 bp) [14]. The effects of these two SNPs are strongly correlated (r > 0.8), which indicates that they may be in strong LD with the same underlying causal mutation with pleiotropic effects: i.e. simultaneously increasing carcass and skeletal weights and lean meat yield and decreasing dressing percentage, fatness, and muscling (i.e. CEMD), while increasing wool growth.

**Table 7:** Examples of pleiotropic effects of SNPs selected from the multi-trait analyses on the individual traits (signed values with |t| > 1 are shown)

CHR:POS^a^	GFW	YLD	SL	SS	FD	FDC	CU	WR	BC	CC	DAG	SST	WE	CHA	FR	DU	COL	CLZ	CLYZ	CLY	CLX	SK	Gene code
1:244,308,221			2.0	1.3	−5.5	1.1	−1.3			−1.4					−1.3		−2.8		1.3				*TRPC1*
3:37,206,022		−1.5	−4.1	−1.1	−5.5	2.7	1.2		−1.1	1.0		−2.9			1.6		1.1		−1.1				*LCLAT1*
3:39,080,949		2.1	3.2		−5.5		−3.7			1.0		2.7					1.2	−1.9		−2.0	−1.8		*ANTXR1*
3:58,612,068	−1.3	3.1	3.0		5.0	−3.2		−3.1	−1.6					−1.7	−2.6		1.3	−2.2		−2.4	−2.5	−1.5	*KRCC1*
3:59,019,274^(6)^			9.5	2.4	4.1	−3.7	1.1	−7.0	−4.3	−3.9	−1.4				−4.4	2.3			1.1			−4.9	*FOXI3*
3:60,384,564	3.5		−5.0			2.7	−2.5	5.6	1.8	1.2					3.3	−2.8	1.9		1.2			3.9	*TTL*
3:133,925,825	−1.4	1.6			−1.6	−1.3		−2.5	−1.0	−1.4		−2.9		−4.6	−1.5		−1.4					−1.7	*KRT86*
3:137,105,001	3.5		1.4					1.4				2.0	−1.3	1.4								1.2	*WNT1*
3:202,672,118	−2.0	−2.5			6.1	−2.6	3.0		−2.4	−3.2		1.9		2.3		1.3		−2.3	1.1	−2.2	−2.3		
4:62,652,234		−1.4	−1.8		−2.4	1.4	2.7	1.8				−1.7		−2.4				5.1	−3.0	4.5	4.5		*BMPER*
5:48,528,440^(3)^	2.8	2.2	3.3		2.0	1.3	−3.2	−1.3			−1.6		2.0				2.7	2.9	−1.1	2.7	2.8		*FTL*
6:37,256,712^(4)^	4.4		−2.3	−1.5		3.6	−1.8					1.6		3.5	3.2		1.0	−1.1					*NCAPG/LCORL*
6:86,591,198	−1.1	1.3	1.2		−1.5	1.8		−2.4		2.7			2.0	3.5	3.0			−1.3		−1.3	−1.4	−1.4	*GC*
6:94,602,390			−3.7		−3.7		1.2		1.5			−2.2					−3.0		−1.1				*FGF5*
6:114,171,155	1.2				2.8			−1.5	−1.2		1.1		−1.4				−1.6	−5.3	2.5	−4.8	−4.8		*TRMT44*
7:57,834,812	3.4	1.5	1.8	−1.3	−2.2		−1.1	−1.5	−1.2	−1.3			1.4	1.2		1.0	1.7	−1.0		−2.1	−1.5		*FGF7*
8:31,242,591^(5)^	−2.4	−3.1	−1.3	−1.3	−5.5		4.7	−1.3		−1.6	1.1	−1.7			−1.1		−1.5	1.3	−2.3				
11:26,333,173	5.0	−1.6	1.0	−2.0	−1.9	2.7	−2.6		−1.1								1.5						*ALOX15*
11:49,956,916	4.5	2.1	2.1				−5.5	1.2	−1.0	1.3	2.3			−1.3									*FASN*
13:22,846,602				−2.2	−1.3				1.3	5.3	1.9	−1.0					−2.5						*PIP4K2A*
13:62,872,216^(6)^		−2.5	−3.8	1.8	−4.1	−5.5	1.7			1.1		−3.3	−2.7	−5.3				2.2		2.1	2.0		*RALY*
14:55,112,107^(2)^	−1.5	1.1	−1.1	1.1	−5.5	−4.6	2.3		−2.6			−3.3	−1.2	−1.6					−1.5				*BCL2L12*
15:13,764,013^(2)^		1.0	1.1	−3.3			−5.0			1.0			1.8				1.8	−2.0		−1.7	−1.6		*MAML2*
15:72,565,587			−1.5		−6.6	−1.3	−1.2		−1.0	−1.5		−2.8	−1.5	−2.7	1.3	−2.0	−1.3	2.7	−1.7	2.3	2.3		*ALX4*
19:658,455^(3)^	5.3	−11.3	3.3		2.3	−2.2	−2.1			−1.1	1.1	−3.9	−1.3	−3.6	−2.0	−2.0	−2.8	−1.2	1.7			−2.3	*LANCL2*
21:5,529,725	2.0	1.0			1.3	2.2	−4.9			1.6		−2.2	1.8					−1.2		−1.2	−1.2	−3.4	*NOX4*
22:34,770,924	1.4	1.2		−2.1	−4.3			−2.6		−1.6	1.4	1.2	1.3	1.4		2.4	2.1			−1.1	−1.0		*ATRNL1*
23:44,250,113^(5)^	−2.2		1.2	2.2	2.4	−5.3		−1.9	−2.4	−4.3	−3.1		1.6					−2.2		−2.2	−2.3		
24:35,659,971					−1.7		1.0	2.9		1.3	1.1						−1.5	4.1		5.1	5.1	1.1	*ACHE*
25:35,306,299^(11)^	−3.8	−4.0	−3.1	−8.7	−3.8	6.4	4.4		1.2	3.4	−1.8			2.9		1.3		2.1	−2.9				*MAT1A*
26:22,464,394			2.4		5.5					−1.1	1.1	3.7	1.3					−2.1	2.2	−1.6	2.0		*DLC1*

Traits at yearling age: FDC = FDCV; CU = CURV; WR = BRWR; BC = BCOV; CC = CCOV; SST = SSTRC, WE = WEATH, CHA = CHAR; FR = FLROT; DU = DUST; COL = GCOL; CLZ = COLZ; CLYZ = COLYZ; CLY = COLY; CLX = COLX; SK = SKINQ

^a^Ovine chromosome and position of SNPs, and the superscript numbers in brackets are the number of SNPs that have a similar pattern of pleiotropic effects within a distance of 1 to 1.5 Mb

In a single-trait GWAS with the 50 K SNP chip in Chinese Merino sheep, Wang et al. [55] identified 28 SNPs that affect fibre diameter, fibre diameter coefficient of variance, and crimp and are located within 12 genes. However, we did not find any significant SNPs (*P* < 10^−5^) within these genes.

### Application to wool quality improvement

The patterns of the effects of the QTL that we studied here indicate that they have various degrees of usefulness for selection. Some QTL have an allele with desirable effects on more than one trait and appear to be good targets for selection. For instance, the QTL on OAR11 (located at the edge of the *FASN* gene) has an allele that increases greasy fleece weight, wool yield, and staple length and decreases fibre diameter. This pattern of quality (sheep with higher fleece, higher yield, longer staples, and finer wool) is desirable for sheep breeders and the wool industry because these traits affect the price paid for wool to the producer and the processing efficiency and use of the wool in manufacturing. A SNP on OAR23 at about 44.2 Mb had an allele that increases staple strength and decreases fibre diameter coefficient of variance, breech wrinkle, breech cover, crutch cover and dag, which is a valuable pattern for resistance to breech strike and would reduce the need for flystrike prevention strategies such as mulesing and chemical treatment. SNPs on OAR3:137.1 Mb (*KRT86*), OAR3:133.9 Mb (*WNT1*), OAR8:89.0 Mb (*MPC*), and OAR12:49.9 Mb (*RSC1A1*) had an allele associated with better staple structure (staple with very fine bundles) and wool character (well-defined crimp with low variation along the staple), which is a desirable pattern for wool manufacturing. Generally, for a given trait, SNPs showed a similar association at both of the ages at which the trait was measured. However, none of the associations explained a large fraction of the variance for any trait. Therefore, although incorporation of the identified QTL may not increase the accuracy of single-trait EBV, they may be useful to manage unfavourable genetic correlations between traits.

## Conclusions

For many wool traits, accuracy of genomic prediction was low (average over all traits = 0.22), especially for traits with a low heritability, few records and for which few QTL were identified. In an attempt to identify more QTL for these traits, we examined three approximate multi-trait methods. As well as a multi-trait GWAS, we describe two new multi-trait methods based on single-trait BayesR results. Collectively, these three methods mapped 206 putative QTL of which 20 were common to all methods. Sixteen genes that are located near a significant SNP have known effects on hair growth and a further five significant SNPs are near genes that were previously reported for QTL for growth and body composition. Future research should examine whether genomic prediction accuracy can be increased by using the QTL identified in this paper.

## Additional file

**Additional file 1: Table S1.** Pleiotropic effects of 206 SNPs selected from multi-trait analyses on individual traits. The signed significant t-values are provided for 206 SNPs across 22 traits at two ages. Gene names are also provided if SNPs were located in or within a distance of 50 kb of the gene.
